# Polyphyletic origin of MERS coronaviruses and isolation of a novel clade A strain from dromedary camels in the United Arab Emirates

**DOI:** 10.1038/emi.2016.129

**Published:** 2016-12-21

**Authors:** Susanna K P Lau, Renate Wernery, Emily Y M Wong, Sunitha Joseph, Alan K L Tsang, Nissy Annie Georgy Patteril, Shyna K Elizabeth, Kwok-Hung Chan, Rubeena Muhammed, Jöerg Kinne, Kwok-Yung Yuen, Ulrich Wernery, Patrick C Y Woo

**Affiliations:** 1 Department of Microbiology, The University of Hong Kong, Hong Kong, China; 2 State Key Laboratory of Emerging Infectious Diseases, Research Centre of Infection and Immunology, Carol Yu Centre for Infection, Collaborative Innovation Center for Diagnosis and Treatment of Infectious Diseases, The University of Hong Kong, Hong Kong, China; 3 Central Veterinary Research Laboratory, PO Box 597, Dubai, UAE

**Keywords:** clade A, dromedary camels, MERS, Middle East respiratory syndrome coronavirus, novel, polyphyletic, United Arab Emirates

## Abstract

Little is known regarding the molecular epidemiology of Middle East respiratory syndrome coronavirus (MERS-CoV) circulating in dromedaries outside Saudi Arabia. To address this knowledge gap, we sequenced 10 complete genomes of MERS-CoVs isolated from 2 live and 8 dead dromedaries from different regions in the United Arab Emirates (UAE). Phylogenetic analysis revealed one novel clade A strain, the first detected in the UAE, and nine clade B strains. Strain D998/15 had a distinct phylogenetic position within clade A, being more closely related to the dromedary isolate NRCE-HKU205 from Egypt than to the human isolates EMC/2012 and Jordan-N3/2012. A comparison of predicted protein sequences also demonstrated the existence of two clade A lineages with unique amino acid substitutions, A1 (EMC/2012 and Jordan-N3/2012) and A2 (D998/15 and NRCE-HKU205), circulating in humans and camels, respectively. The nine clade B isolates belong to three distinct lineages: B1, B3 and B5. Two B3 strains, D1271/15 and D1189.1/15, showed evidence of recombination between lineages B4 and B5 in ORF1ab. Molecular clock analysis dated the time of the most recent common ancestor (tMRCA) of clade A to March 2011 and that of clade B to November 2011. Our data support a polyphyletic origin of MERS-CoV in dromedaries and the co-circulation of diverse MERS-CoVs including recombinant strains in the UAE.

## INTRODUCTION

Since its first appearance in 2012, Middle East respiratory syndrome (MERS) has affected more than 1300 cases in more than 25 countries in four continents, and has an alarming fatality rate of more than 30%.^1^ A novel lineage C betacoronavirus, MERS coronavirus (MERS-CoV), has been confirmed to be the etiological agent of MERS.^2, 3^ Subsequent detection of MERS-CoV and its antibodies in dromedaries in various countries in the Middle East and North Africa has suggested that these animals are probably the reservoir for MERS-CoV.^4, 5, 6^ In addition, both before and after the MERS epidemic, the discovery of other closely related lineage C betacoronaviruses in various bat species and hedgehogs suggests that these animals may be hosts for an ancestor of MERS-CoV.^7, 8, 9, 10^ In further support of this hypothesis, the spike protein of *Tylonecteris* bat CoV HKU4 binds dipeptidyl peptidase 4,^11, 12^ the cellular receptor for MERS-CoV.

As of August 2016, 212 genome sequences of MERS-CoV were available in GenBank. Although 91 of the sequences were obtained from MERS-CoV in dromedaries, a large proportion of them were from a recent study in Saudi Arabia.^13^ The small number of dromedary MERS-CoV genomes obtained from other countries has hindered understanding of the epidemiology and evolutionary history of the virus in camels outside of Saudi Arabia. In our previous report on MERS-CoV epidemiology in Dubai, nine dromedary MERS-CoV strains were sequenced and found to be closely related.^14^ Recently, we have reported another dromedary MERS-CoV strain detected in an isolated dromedary herd in the United Arab Emirates (UAE).^15^ Complete genome sequencing and phylogenetic analysis has indicated that this MERS-CoV strain is a unique member of a cluster of closely related MERS-CoV strains obtained from patients in the Hafr-Al-Batin region of Saudi Arabia and Qatar,^16, 17^ as well as those from patients in the recent Korean outbreak.^18^ From the results of these two studies, we hypothesized that diverse MERS-CoV strains may be circulating in dromedaries of the UAE. To test this hypothesis, we performed complete genome sequencing of 10 additional strains of MERS-CoV isolated from dromedaries in different regions of the UAE. The results support a polyphyletic origin of MERS-CoV in dromedaries and the co-circulation of diverse strains from multiple sources in the same farm. A novel clade A strain, the first identified in the UAE, may belong to a separate lineage, A2, circulating in dromedaries.

## MATERIALS AND METHODS

### Strains and viral culture

Ten MERS-CoVs isolated from 10 respiratory samples from 10 dromedaries sent to the Central Veterinary Research Laboratory in Dubai, UAE, in 2014 and 2015 were included in this study. Isolation of MERS-CoV was performed as previously described.^15^ Briefly, the samples were diluted 10-fold with viral transport medium and filtered. Two hundred microliters of the filtrate was inoculated into 200 μL of Minimum Essential Medium (Gibco, Grand Island, NY, USA). Four hundred microliters of the mixture was added to 24-well tissue culture plates containing Vero cells for adsorption inoculation. After 1 h of adsorption, excess inoculum was discarded, the wells were washed twice with phosphate-buffered saline, and the medium was replaced with 1 mL of Minimum Essential Medium (Gibco). The cultures were incubated at 37 °C with 5% CO_2_ and were inspected daily by inverted microscopy for cytopathic effect (CPE) for five days. Each of the cultures exhibited the typical CPE of detachment and rounding of cells. All cultures with a CPE were confirmed to be infected with MERS-CoV through a real-time quantitative RT-PCR assay targeting a region of the viral genome upstream of the envelope gene and isothermal amplification with a Genie instrument (Optigene Limited, Horsham, UK).

### RNA extraction

Viral RNA was extracted from the cultures using a QIAamp Viral RNA Mini Kit (Qiagen, Hilden, Germany). The RNA was eluted in 60 μL of Buffer AVE and was used as the template for RT-PCR.

### Complete genome sequencing

The complete genomes of the 10 isolated dromedary MERS-CoV strains were sequenced as previously described.^14, 15^ Briefly, the RNA extracted from the MERS-CoV strains was converted to cDNA by using a combined random priming and oligo(dT) priming strategy. The cDNA was amplified by primers designed on the basis of multiple sequence alignments of available MERS-CoV genome sequences using previously described strategies.^19^ The 5′ ends in the viral genomes were confirmed via rapid amplification of cDNA ends (RACE) using a 5′/3′ RACE kit (Roche Diagnostics GmbH, Mannheim, Germany). Sequences were assembled and edited to produce the final sequences of the MERS-CoV genomes.

### Genome analysis

Nucleotide and amino acid sequences of predicted open reading frames (ORFs) and the full genomes of 10 MERS-CoV were aligned with 85 human MERS-CoV and 87 camel MERS-CoV genomes using Multiple Alignment using Fast Fourier Transform (MAFFT). Ten MERS-CoV genome sequences from GenBank were not included in the analysis because of an incomplete 5′ genomic region or having redundant sequences of a single strain. Pairwise identity of the 182 MERS-CoV genome sequences as well as their ORFs and predicted proteins was calculated using MEGA5.

### Phylogenetic analysis

Maximum-likelihood phylogenetic trees with 1000 bootstrap replicates were constructed using PhyML v3.0 (Montpellier, France) on the basis of the complete genome, ORF1a, ORF1b, and S genes of 85 human and 97 camel MERS-CoV genomes. The best-fit substitution model was selected using jModelTest and used in the maximum-likelihood analysis.

### Recombination analysis

To detect possible recombination, bootscan analysis was performed by using the nucleotide alignment of the genome sequences of MERS-CoV and Simplot version 3.5.1, as previously described.^20, 21^ The analysis was conducted using a sliding window of 1500 nucleotides moving in 200 nucleotide steps with genome sequences obtained in the present study as the query. Possible recombination sites suggested by the bootscan analysis were confirmed through multiple sequence alignments.

In addition to the bootscan analysis, possible recombination breakpoints were also detected using RDP, GENECONV, BOOTSCAN, MAXIMUM CHI SQUARE, CHIMAERA, SISCAN and 3Seq implemented in Recombination Detection Program Version 4 (RDP4). Automasking was used for optimal recombination detection. The RDP analysis was run without a reference and with a window size of 60, the BOOTSCAN window size was increased to 500, the MAXCHI and CHIMAERA number of variable sites per window was increased to 120, and the window size and step size for SISCAN were increased to 500 and 20, respectively. Potential recombination events detected by four or more of the seven independent recombination detection methods in the ten genomes in this study were further analyzed with phylogenetic trees constructed using sequences upstream and downstream of the potential recombination breakpoint.

### Estimation of divergence times

Divergence times for the MERS-CoV strains were calculated using a Bayesian Markov chain Monte Carlo (MCMC) approach implemented in BEAST (version 1.8.0), as described previously.^22, 23^ One representative strain was selected for MERS-CoV strains with close sequence similarity and obtained from the same outbreak. Analyses were performed under the SRD06 substitution models for the concatenated main coding regions of the genome (ORF1ab, S, E, M and N), with an uncorrelated lognormal molecular clock and a GMRF skyride coalescent model. The MCMC run was 1 × 10^8^ steps long with sampling every 1000 steps. Convergence was assessed on the basis of the effective sampling size after a 10% burn-in using Tracer software, version 1.6.0.^22^ The mean time to the most recent common ancestor (tMRCA) and the highest posterior density regions at 95% (HPDs) were calculated. The trees were summarized in a target tree by using the Tree Annotator program included in the BEAST package by choosing the tree with the maximum sum of posterior probabilities (maximum clade credibility) after a 10% burn-in.

### Nucleotide sequence accession numbers

The nucleotide sequences of the ten dromedary MERS-CoV genomes sequenced in this study have been submitted to the GenBank sequence database under accession nos. KX108937–KX108946.

## RESULTS

### Clinical and epidemiological data

**Figure 1:**
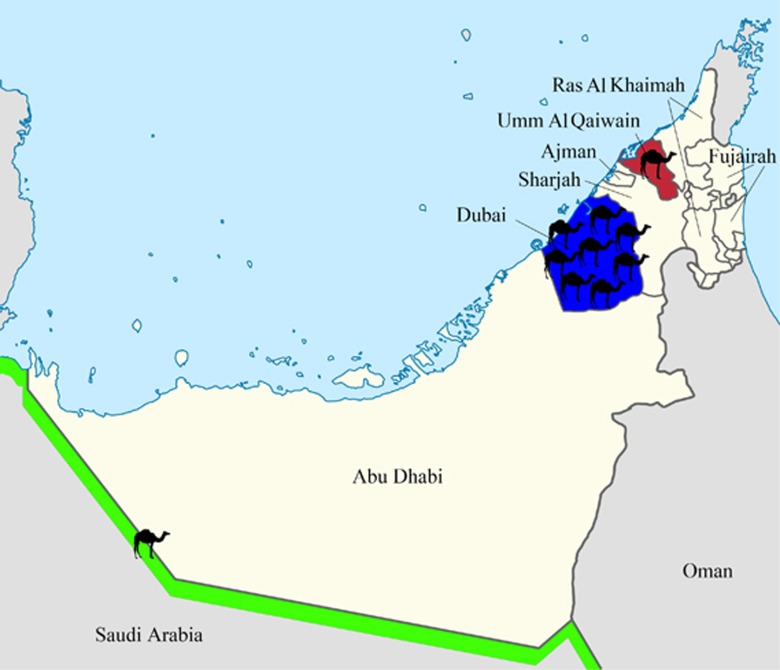
Geographical distribution of dromedaries in the United Arab Emirates infected with MERS-CoV strains that were isolated in the present study. The number of dromedaries corresponds to the number of MERS-CoV isolates from the sampling location.

We isolated 10 MERS-CoV strains from nasal swabs of two live and eight dead dromedary calves from different regions in the UAE, including a breeding herd, which returned from its winter pasture in Saudi Arabia and calves from dromedary dairy farms in Dubai and Umm Al Quwain. The clinical and epidemiological data of the 10 dromedaries infected with MERS-CoV strains isolated and sequenced in this study are summarized in Table 1 and Figure 1. The median age of the dromedaries was 1 month (range: 15 days to nine months). Two of the eight dead dromedaries exhibited nasal discharge before they died.

### Phylogenetic analysis

**Figure 2:**
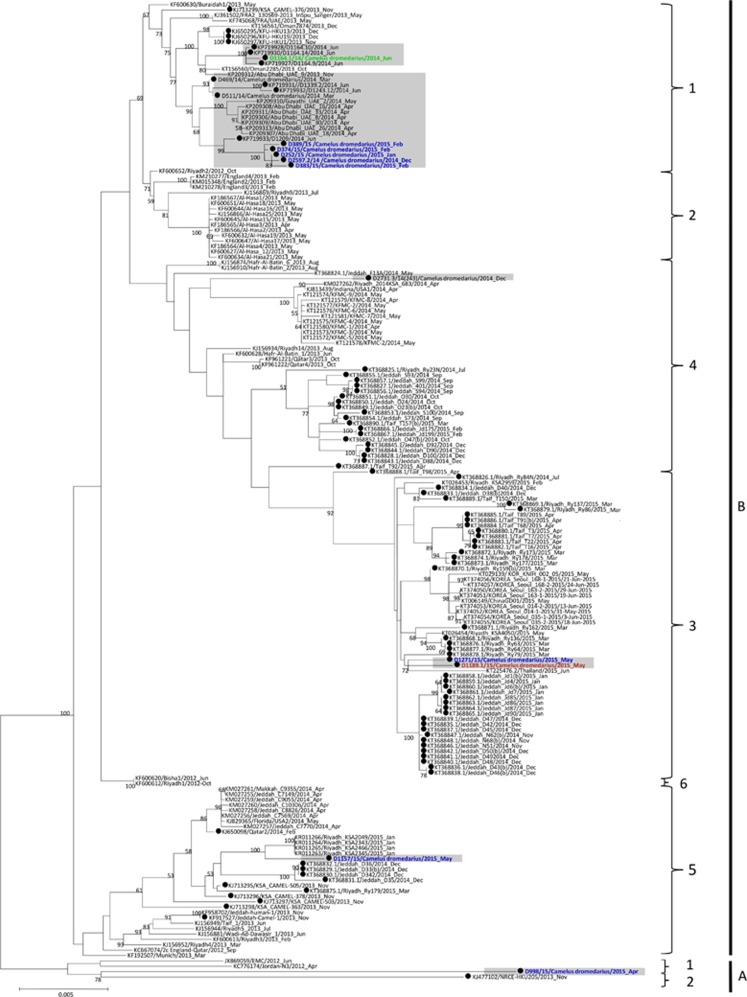
Maximum-likelihood phylogeny based on the complete genome sequences of 182 MERS-CoV strains. A general time-reversible model of nucleotide substitution with estimated base frequencies, the proportion of invariant sites, and the gamma distribution of rates across sites were used in the maximum-likelihood analysis. Bootstrap values are shown next to the branches. The scale bar indicates the number of nucleotide substitutions per site. MERS-CoVs from dromedaries are indicated in black circles. The ten MERS-CoV strains sequenced in the present study are colored: blue, Dubai; brown, Umm Al Quwain; green, Saudi Arabia. MERS-CoVs isolated from the United Arab Emirates are indicated by gray boxes.

**Figure 3:**
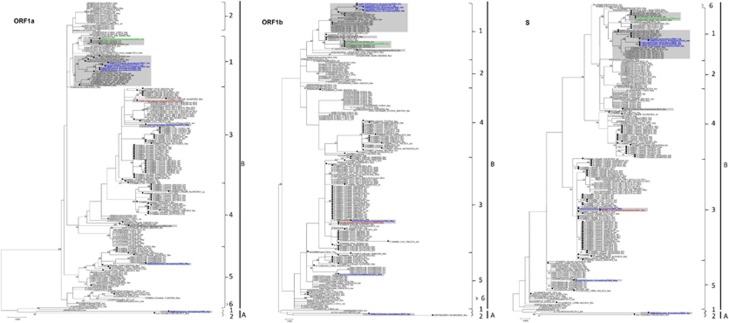
Maximum-likelihood phylogeny based on ORF1a, ORF1b and S gene sequences of 182 MERS-CoV strains. TIM1+I+G, GTR+I+G, and TK 2+I substitution models were selected for the ORF1a, ORF1b and S gene trees, respectively. Bootstrap values are shown next to the branches. The scale bar indicates the number of nucleotide substitutions per site. MERS-CoVs from dromedaries are indicated in black circles. The 10 MERS-CoV strains sequenced in the present study are colored: blue, Dubai; brown, Umm Al Quwain; green, Saudi Arabia. MERS-CoVs isolated from the United Arab Emirates are indicated by gray boxes.

Phylogenetic analysis of the complete genomes of the ten sequenced MERS-CoV isolates showed that one isolate belongs to clade A, and nine belong to clade B (Table 2; Figure 2). Phylogenetic analysis of the complete genome, ORF1a, ORF1b and the S gene sequences all showed that the clade A strain, D998/15, isolated from a 1-month-old dromedary calf from a special dairy farm in Dubai, is a unique member of clade A, being most closely related to another dromedary isolate, NRCE-HKU205, which was previously detected in Egypt (Figure 3). Moreover, these two isolates formed a separate cluster distinct from the other two known clade A strains, EMC/2012 and Jordan-N3/2012, both isolated from humans. The results suggest that clade A isolates from humans and camels may form two separate lineages, A1 and A2. This hypothesis is further supported by analysis of amino acid substitutions along the whole genome sequences.

Comparison of deduced amino acid sequences of proteins among clade A1, clade A2 and the six lineages of B strains showed a total of 16 substitutions, most occurring in the viral nsp3 and S proteins (Table 2). Notably, two substitutions in nsp3, at positions 1574 and 1717, were found within the catalytic domain of PL^pro^.^24^ Highly variable residues were observed at position 1574, which has been reported to be under positive selection.^25^ Another substitution in the S protein at position 1020 (glutamine in clade A strains and histidine/arginine in clade B strains) was found within heptad repeat 1 of the S2 region. Interestingly, the heptad repeat region has recently been reported to be a major selection target among MERS-CoVs, and different residues at position 1020 may affect the stability of the six-helix bundle formed by the heptad repeats.^26^ There were nine amino acid substitutions between clade A and B strains, and five amino acid substitutions between clade A1 and A2 strains. From these results, we propose that the two clade A dromedary isolates D998/15 and NRCE-HKU205 should be classified under a new lineage, A2, which is distinct from the two clade A human isolates EMC/2012 and Jordan/2012.

The nine clade B isolates belong to three distinct lineages (Figure 2). Five isolates from a dairy farm in Dubai and one from a Dubai breeding herd returning from Saudi Arabia belong to lineage 1. One isolate from a dairy farm in Dubai and one from Umm Al Qaiwain belong to lineage 3. One isolate from a dairy farm in Dubai belongs to lineage 5 (Figure 2). Phylogenetic trees based on ORF1a, ORF1b and the S gene were further constructed to assess strains that had an inconsistent phylogenetic position in the different trees. The two lineage three strains in this study (D1271/15 and D1189.1/15), along with other lineage three strains, were found to cluster more closely with lineage 4 MERS-CoV in the tree constructed for ORF1a, but cluster more closely with lineage 5 MERS-CoV in the tree constructed for ORF1b (Figure 3). For the tree constructed for the S gene, these lineage three strains did not cluster closely with either lineage 4 or lineage 5 MERS-CoV (Figure 3). The remaining eight strains identified in this study were found to consistently cluster within the same lineages in all three trees (Figure 3).

### Recombination analysis

**Figure 4:**
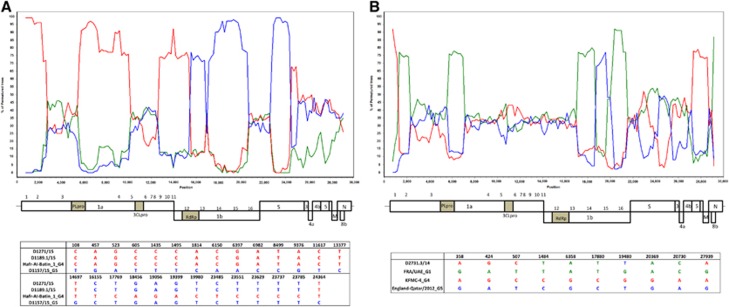
Detection of potential recombination events by bootscan analysis and multiple alignments. Bootscanning was conducted with Simplot version 3.5.1 (F84 model; window size, 1,500 bp; step, 200 bp) on a gapless nucleotide alignment. (**A**) D1271/15 and D1189.1/15 were used as the query sequences and compared with the genome sequences of a lineage 4 MERS-CoV strain Hafr-Al-Batin 1 (red, KF600628), a lineage 1 MERS-CoV strain UAE/Abu Dhabi_UAE_9 (green, KP209312) and a lineage 5 MERS-CoV strain D1157/15 (blue, KX108944). (**B**) D2731.3/14 was used as the query sequence and compared with the genome sequences of a lineage 1 MERS-CoV strain, FRA/UAE (green, KF745068), a lineage 4 MERS-CoV strain, KFMC-4 (red, KT121575) and a lineage 5 MERS-CoV strain, England-Qatar/2012 (blue, KC667074).

Because phylogenetic analyses showed inconsistent phylogenetic positions for strains D1271/15 and D1189.1/15 in the ORF1a and ORF1b trees, we performed bootscan analysis and multiple sequence alignments for these two strains. For both strains, bootscan analysis showed high bootstrap support for clustering between the two strains and lineage 4 MERS-CoV in two parts of their genomes (position 1–15000); but for position 15000–24000, bootscan analysis showed high bootstrap support for clustering between the two strains and lineage 5 MERS-CoV (Figure 4A). A multiple sequence alignment using these two strains, a lineage 4 MERS-CoV and a lineage 5 MERS-CoV further indicated that upstream of position 14697, the two strains possessed nucleotides identical to lineage 4 MERS-CoV; but from position 16155–23785, the two strains possessed nucleotides similar to lineage 5 MERS-CoV (Figure 4A).

**Figure 5:**
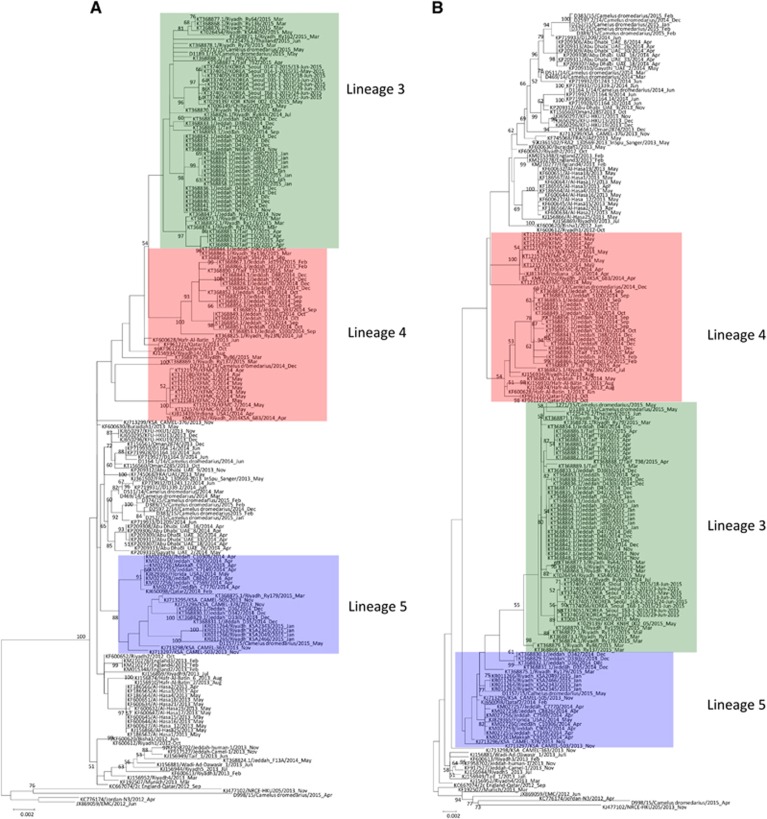
Maximum-likelihood trees constructed using nucleotide sequences of non-recombination regions (**A**), and recombination regions (**B**) detected using RDP4. The non-recombination region is approximately positions 1 to 14957 and 28836 to 30087, and the recombination region is approximately positions 14958 to 28835, with position numbering based on the D1189.1/15 genome sequence. Lineages are indicated by colored boxes: lineage 1, green; lineage 3, red; and lineage 5, blue.

For the analysis using RDP4, recombination breakpoints were detected in the two genomes at positions 14958 and 28835. Phylogenetic trees constructed using concatenated nucleotides sequences from position 1 to 14957 and 28836 to 30087, and from position 14958 to 28835 confirmed the inconsistent phylogenetic position of these two newly detected strains in the two trees (Figure 5).

### Estimation of divergence dates

**Figure 6:**
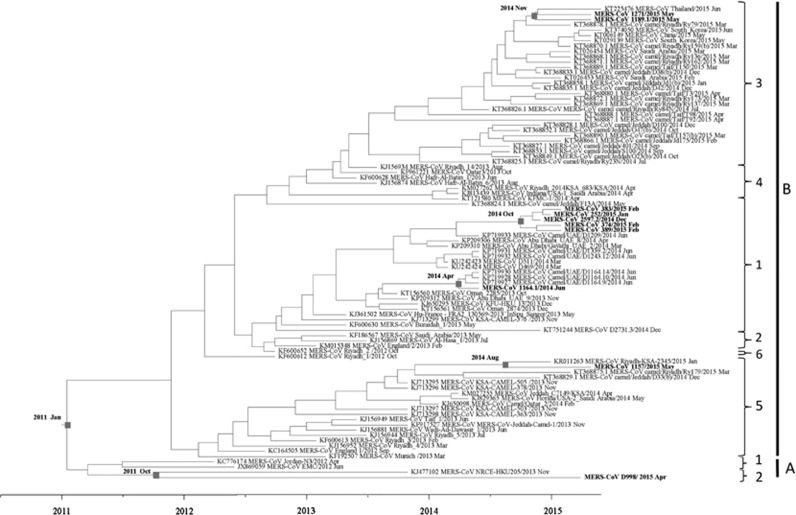
Estimation of time to the most recent common ancestor for MERS-CoV. The time-scaled phylogeny was summarized from all Markov chain Monte Carlo phylogenies of the concatenated coding regions (ORF1ab, S, E, M and N) of 90 phylogenetically distinct MERS-CoV genomes, which were analyzed under the relaxed-clock model with an uncorrelated lognormal distribution in BEAST version 1.8.0. The 10 MERS-CoV genomes sequenced in this study are in boldface.

The inferred evolutionary rate of MERS-CoV was 8.55 × 10^−4^ (95% HPD: 7.09 × 10^−4^, 1.01 × 10^−3^). The divergence time of the present 10 dromedary strains is shown in Figure 6. On the basis of time-resolved phylogeny, the root of the tree is January 2011 (95% HPD: June 2010, August 2011). The time of the most recent common ancestor (tMRCA) of clade A was dated back to March 2011 (95% HPD: August 2010, September 2011) and that of clade B to November 2011 (95% HPD: July 2011, March 2012) (Figure 6).

## DISCUSSION

In this study, we showed that MERS-CoVs isolated from dromedaries in the UAE were of polyphyletic origin. For the eight MERS-CoV strains isolated from dromedaries from dairy farms in Dubai, one belongs to clade A (D988/15) and seven (D2597.2/14, D252/15, D374/15, D383/15, D389/15, D1157/15 and D1271/15) belong to clade B (Figure 2). Interestingly, the source clade A strain, D988/15, was isolated from a young female dromedary in a special dairy farm with camel mothers from Pakistan. Therefore, the source of this virus was most probably different from that of the seven clade B strains, which were isolated from dromedaries from a different dairy farm. The latter farm is a large closed system, which may explain the relative genetic closeness of the circulating MERS-CoV strains. Yet, the seven clade B isolates belonged to three distinct lineages, with five isolates (D2597.2/14, D252/15, D374/15, D383/15 and D389/15) clustered with several other MERS-CoV from Dubai.^27^ For the MERS-CoV isolated from a dromedary calf returning from Saudi Arabia (1164.1/14), the dromedary was from a herd of approximately 50 female breeding dromedaries in Dubai. Every year, these camels were brought to Saudi Arabia for 6 months because of the better grazing conditions due to the higher amount of rainfall in Saudi Arabia than in Dubai. The dromedary calves from this herd were born in Saudi Arabia. Young dromedaries are usually removed from the herd and start their training as racing camels when they are one year old. Although this MERS-CoV was most probably acquired in Saudi Arabia, its genome sequence is closely related to two human MERS-CoV strains from Oman (KT156560) and Abu Dhabi (KP209312), respectively (Figure 2). The MERS-CoV isolated from the dromedary in Umm Al Quwain (D1189.1/15) is closely related to D1271/15 in this study and to a human MERS-CoV strain isolated in Thailand (KT225476) (Figure 2). The present data showed that the genome sequences of the MERS-CoV strains isolated in the UAE were intermingled with other MERS-CoV strains found elsewhere in the Middle East or strains from patients who acquired their infections directly or indirectly in the Middle East. Moreover, diverse MERS-CoVs are co-circulating in dromedaries in the UAE, which derive from different sources.

This polyphyletic origin of MERS-CoV is unique among human CoVs. Such a polyphyletic origin of MERS-CoV in dromedaries is similar to the diverse lineages of SARS-CoV-like viruses in horseshoe bats,^28, 29^ but in contrast to the monophyletic origin of most human SARS-CoV strains, MERS-CoV strains from humans are polyphyletic as a result of multiple camel-to-human transmission events.^30, 31^ According to the existing evidence, a single interspecies transmission event probably occurred from bats to palm civets as the intermediate or amplification host, and then from palm civets to humans before the SARS epidemic in 2003.^28, 32, 33^ The rapid expansion of the epidemic arose after efficient human-to-human transmission of the human SARS-CoV strains. In contrast, MERS-CoV has become endemic in dromedaries of the UAE and other countries in the Middle East, with diverse strains being introduced into the human population. This finding is in line with the overall MERS-CoV phylogeny in the Middle East, with the tMRCA of all MERS-CoVs being dated back to around January 2011, thus suggesting that MER-CoV has emerged in humans relatively recently (Figures 2 and 6). The difference in evolutionary pathways between SARS-CoV and MERS-CoV also provides an explanation for why, MERS—unlike SARS, which disappeared rapidly after civets were segregated from humans by closing wild animal markets in provinces in Southern China—has persisted for more than three years and will probably continue to circulate in dromedaries and humans unless effective vaccines are available. Recently, viruses closely related to human coronavirus 229E (HCoV 229E) have also been found in camels.^34^ However, in contrast to the polyphyletic origin of human and camel MERS-CoVs, the HCoV 229E and related dromedary-derived viruses were each monophyletic, thus suggesting that this endemic human coronavirus may constitute a descendant of camelid-associated viruses.

The clade A isolate D988/15, the first clade A MERS-CoV detected in the UAE, may belong to a separate lineage, A2, within clade A. At present, there are only a few clade A isolates, including the first human MERS-CoV isolated in Saudi Arabia (EMC/2012), another human isolate from Jordan (Jordan-N3/2012), two dromedary strains from Egypt (NRCE-HKU205 and NRCE-HKU270 with partial sequence available only)^35^ and six Nigerian strains (genome not completely sequenced).^36^ Although clade A strains are much less common than clade B strains, our results suggest that diverse clade A MERS-CoVs may be present in various countries in the Middle East and Africa. Both phylogenetic and amino acid substitution analyses showed that the current clade A strains may consist of two distinct lineages: one from humans (A1) and one from camels (A2). Further studies to isolate and sequence more clade A strains in humans and camels may provide insights on the role of clade A strains in MERS-CoV evolution and the potential significance of the observed amino acid changes for host adaptation.

The present results show that recombination events are common among certain strains of MERS-CoV. Recombination is a well-recognized mechanism by which CoVs generate diversity. We have previously described recombination among the three human CoV HKU1 genotypes, and other human or animal coronaviruses,^21, 37, 38^ and other researchers have also found recombination in coronaviruses such as feline coronavirus type II and infectious bronchitis virus.^39^ A number of studies have also reported recombination among MERS-CoVs from different countries.^13, 40^ However, we should be cautious when interpreting possible recombination events solely on the basis of phylogenetic analysis to avoid drawing premature conclusions. For example, by phylogenetic tree analysis, strains D1271/15 and D1189.1/15 in the present study and strain D2731.3/14, which we have reported previously,^15^ showed inconsistent phylogenetic clustering in trees based on different viral genes (Figure 3). However, bootscan analysis and multiple alignments did not reveal evidence of recombination for strain D2731.3/14 (Figure 4B). Only strains D1271/15 (from Dubai) and D1189.1/15 (from Umm Al Quwain) showed evidence of a likely recombination event after stepwise examination using phylogenetic analysis, bootscan analysis and multiple sequence alignments (Figure 4a). It is also important to note that the nucleotide sequences of all MERS-CoV genomes are >99% identical. Therefore, even for the strains with possible recombination, there are only approximately 10 base pair differences upstream and downstream of the potential recombination site between the ‘recombinant' and ‘parent' strains. Although these sequence differences may have arisen through recombination, they may also have resulted from individual nucleotide mutations. Further studies with careful interpretation of recombination analysis results will be important to understand the role of recombination in the emergence and evolution of MERS-CoV.

## Figures and Tables

**Table 1:** Clinical and epidemiological data of the 10 dromedaries with MERS-CoV strains isolated and sequenced in this study

**Strains**	**Clade/lineage**	**Sex/age**	**Date of sample collection**	**Clinical samples**	**Live/dead dromedary (causes of death)**	**Origin of dromedary**
D1164.1/14	B1	F/5 months	6 Jun, 2014	Nasal swab	Live	Breeding herd returning from Saudi Arabia
D2597.2/14	B1	NA/6 months	13 Dec, 2014	Nasal swab	Live	Dairy farm in Dubai
D252/15	B1	F/1 month	30 Jan, 2015	Nasal swab	Dead (white muscle disease, isosporosis abdominal edema disease)	Dairy farm in Dubai
D374/15	B1	M/1 month	12 Feb, 2015	Nasal swab	Dead (colisepticemia)	Dairy farm in Dubai
D383/15	B1	F/1 month	14 Feb, 2015	Nasal swab	Dead (colisepticemia)	Dairy farm in Dubai
D389/15	B1	M/15 days	15 Feb, 2015	Nasal swab	Dead (septicemia)	Dairy farm in Dubai
D998/15	A2	F/1 month	23 April, 2015	Nasal swab	Dead (white muscle disease and clostridiosis)	Dairy farm in Dubai^a^
D1157/15	B5	F/1 month	12 May, 2015	Nasal swab	Dead (septicemia)	Dairy farm in Dubai
D1271/15	B3	M/3 month	29 May, 2015	Nasal swab	Dead (high fever, purulent nasal discharge, pneumonia)	Dairy farm in Dubai
D1189.1/15	B3	F/6-9 months	18 May, 2015	Nasal swab	Dead (nasal discharge)	Umm Al Quwain

Abbreviation: Not available, NA.

a A special dairy farm with camel mothers from Pakistan.

**Table 2:** Comparison of amino acid substitutions between different clades and lineages

**Clade/lineage**	**Species**	**Strain**	**nsp2**		**nsp3**									**nsp4**	**S1**		**S2**	
			**588**	**726**	**1000**	**1024**	**1055**	**1066**	**1370**	**1574**	**1717**	**2114**	**2481**	**2780**	**26**	**194**	**1020**	**1158**
										**CD**	**CD**				**NT**	**NT**	**HR1**	
A/1	Human	EMC/2012	A	K	T	S	P	K	M	G	L	A	F	A	A	Y	Q	S
A/1	Human	Jordan-N3 /2012	A	K	T	S	P	K	M	G	L	A	F	A	A	Y	Q	S
A/2	Camel	D998/15	T	K	T	F	P	E	M	K	L	V	C	A	V	Y	Q	S
A/2	Camel	NRCE- HKU205	A	K	T	F	P	E	M	R	L	V	C	A	V	Y	Q	S
B/5	Human and Camel	All	T	N	I	S	S	K	I	K/E	I	V	F	V	V	H	H/R	A
B/6	Human and Camel	All	T	N	V	S	S	K	I	K	I	V	F	V	V	H	R	A
B/4	Human and Camel	All	T	N	V	S	S	K	I	K/G/E	I	V	F	V	V	H	R	A
B/3	Human and Camel	All	T	N	V	S	S	K	I	K/G	I	V	F	V	V	H	R	A
B/2	Human and Camel	All	T	N	V	S	S	K	I	K	I	V	F	V	V	H	R	A
B/1	Human and Camel	All	T	N	V	S	S	K	I	K/E	I	V	F	V	V	H	R	A

Abbreviations: Catalytic domain, CD; N-terminal, NT; heptad region 1, HR1.
